# Peer Review of “SARS-CoV-2 Vaccination Uptake in a Correctional Setting: Cross-sectional Study”

**DOI:** 10.2196/31905

**Published:** 2021-09-28

**Authors:** Benjamin A Howell

**Keywords:** vaccination, COVID-19, incarcerated individuals, correctional facility, public health, pandemic, vaccine, carceral setting, vaccine implementation, correctional staff

*This is a peer-review report submitted for the paper “SARS-CoV-2 Vaccination Uptake in a Correctional Setting”.*

## Round 1 Review

### General Comments

This is an important manuscript [1] describing the efforts of the Rhode Island Department of Corrections (RIDOC) to roll out a vaccine program in their unified state correctional system. First, I would be careful in describing this as an “evaluation.” It is a description of the rollout of the vaccine program, and I did not find any elements of an evaluation. Second, the manuscript could be much improved with increased clarity in the writing. Even as a reader who knows more about the RIDOC correctional system than the average reader, I got confused at times about what the authors were referring to. Adding more details on the RIDOC (and how it compares to other correctional systems) will aid generalizability, and also adding more details about the RIDOC vaccination program will help readers contextualize their findings. I recommend rewriting this manuscript with a more general public health audience in mind (who will likely know less about correctional systems).

### Specific Comments

#### Major/Minor Comments

##### Introduction

1. “Correctional outbreaks have substantially contributed…” While I agree that is likely true, this statement relies on the citation of one study that describes county-level infection rates based on infection rates in one (very large) county jail. I would hedge more with the language here as done in the Discussion section.

2. “The goal of this study was to evaluate…” As mentioned above, I would not call this an “evaluation” per se. Even as a largely descriptive piece, the data reported here are important, so I do not think the authors need to oversell it as an “evaluation.” Evaluation implies that they attempted to figure out differences in vaccine acceptance rate or why/how the program worked/did not work or something like that. There is none of that here.

##### Methods

1. “From the beginning…” What makes RIDOC procedures around testing and isolation “aggressive”?

2. “The RIDOC is…” First, the authors should be consistent using “RIDOC” or “The RIDOC.” Second, I think this is where more work can be done to explain the RIDOC to general public health audiences. For example, the term “security facilities” will likely be opaque to many. Third, the authors used “sentenced…individuals” here, but in the next sentence refer to the same people as “incarcerated people who had received a sentence after a criminal trial,” which is confusing.

3. “This includes individuals…” This is vague, and I believe this is included to make the results from the RIDOC generalizable to other states, but more clarification of why this sentence is included would be helpful. The authors could also use this section to describe where a typical “jail” population is housed in the RIDOC system (ie, the Intake facility).

4. “Among incarcerated people, a general system of “Rounds”…” Is “Rounds” here a synonym for what is described as “phases” in the next paragraph? It is unclear what is meant by this term. Also, they should be clearer about what they mean by “opt-out.” More descriptions of how this process was rolled out will be helpful for readers hoping to implement similar programs. How did they approach individuals who were incarcerated? What education was provided?

5. More details in the paragraph on phases would be helpful, for example, in the sentence beginning “In phase 2...” If more description of the RIDOC facilities is given, they can refer to that here. To people unfamiliar with the RIDOC, what a “smaller facility” means would be confusing. This is also the case with the next sentence and the reference to “Medium Security.”

6. “Among corrections staff…” As above, I think being clearer about what is meant by “opt-in” here would be helpful, especially as it contrasts with the “opt-out” system described for incarcerated individuals.

##### Results

1. The sentence on influenza does not need to be in parentheses.

2. “…declined the offer of vaccine.” This is awkward—may be missing an indefinite/definite article or needs to be phrased differently (ie, “declined the offer of a vaccine” or “declined to be vaccinated”).

3. “A total… did not opt-in for the initial vaccine offering.” The authors mean “did not opt in for a vaccine during the initial vaccine offering,” not opting in for the vaccine offering.

4. “Due to logistics…due to vaccine delivery times and staffing availability.” The sentence is awkwardly structured. The “logistics” are the “delivery times and staffing availability,” or are they referring to something else?

5. “At the time…” The reference to the Intake facility will be confusing to people who are not aware of the structure of the RIDOC. The authors may want to flag “Intake” as being equivalent to a “jail” population in other states that has a mix of “awaiting-trial” and “sentenced” individuals. If the Intake facility only has awaiting-trial individuals, this should be clearer. As it is referred to here, it is vague and confusing.

##### Discussion

1. “Vaccination was efficient…” What about it was efficient? I think the authors mean that they vaccinated 70% of the population within 4 months, but this should be more explicitly stated if that is what they meant.

2. “This aligns…” I would break this sentence into two sentences. There are two important points being made here and they should highlight both: (1) the RIDOC is on target to achieve herd immunity and (2) concerns about vaccine hesitancy in incarcerated populations may be overstated. Also, this second point makes a description of how they structured the education and approach to incarcerated individuals that much more important.

3. “The pandemic has devastated correctional settings…” This sentence is awkward, and the use of devastated needs to be qualified (as is, it feels too subjective).

4. “Similarly…” This sentence is awkward. The authors mean to say that both mass incarceration and COVID-19 have disproportionately impacted communities of color, but they should say it more clearly. Also, they should be consistent using “Covid-19” or “COVID-19.”

#### Tables

1. Table 1: Alignment of the “group description” here and what is described in the text. For example, phase 2 here refers to the specific facility but in the text, these are referred to as “smaller facilities,” requiring the reader to make this logical connection. Phase 3 here includes “individuals awaiting transfer,” which is not referred to in the text.

2. Table 1: There is no attached asterisk to where the footnote is referring to.

3. Table 2: It should be made clearer that general individuals in the Intake facility (as being an awaiting-trial population) were not included in the vaccine rollout phases (or was this not true?).

4. Table 2: The population on what day? These populations probably change every day (or even every hour). The authors should flag this in the title of the table when these numbers were collected.

## Round 2 Review

### General Comments

The authors have addressed all my concerns with this revision of their manuscript. My only suggestion is to rereview for typographical or grammatical errors. This revision introduced a couple of errors that I think should be fixed before this paper is fully accepted. For example: in the Abstract/Objective section, “...to describe the a state-wide vaccination...” and in the first sentence of the second paragraph of the Introduction, “From the beginning of the pandemic, the Rhode Island collaborated...”
